# The correlation between blood urea nitrogen to creatinine ratio and short-term and long-term all-cause mortality in sepsis-associated acute kidney injury patients aged 50 and above: a large retrospective cohort study

**DOI:** 10.3389/fendo.2026.1704657

**Published:** 2026-03-27

**Authors:** Bin Yang, Ke Qin, Tingyuan Zhang, Huanzhang Shao

**Affiliations:** 1 Department of Critical Care Medicine, Henan Provincial People’s Hospital, Zhengzhou, Henan, China; 2 Department of Critical Care Medicine, People’s Hospital of Zhengzhou University, Zhengzhou, Henan, China; 3 Henan Key Laboratory for Critical Care Medicine, Zhengzhou, Henan, China

**Keywords:** all-cause mortality, blood urea nitrogen to creatinine ratio, prognostic indicator, retrospective cohort study, sepsis-associated acute kidney injury

## Abstract

**Objective:**

To investigate the association between the blood urea nitrogen to creatinine ratio (BUN/Cr) and all-cause mortality in sepsis-associated acute kidney injury (SA-AKI) patients aged ≥ 50 years.

**Methods:**

This single-center, retrospective cohort study included 764 patients aged ≥ 50 years with SA-AKI hospitalized at Henan Provincial People’s Hospital from January 2020 to August 2024. Patients were grouped into BUN/Cr tertiles: T1 (≤ 16.00), T2 (16.01–22.50), and T3 (> 22.50). The primary outcomes were 28-day, 90-day, and 1-year all-cause mortality. Associations between BUN/Cr and mortality were evaluated using Cox regression and subgroup analyses. Predictive value and dose-response relationships were assessed via receiver operating characteristic (ROC) curves and restricted cubic spline (RCS) models.

**Results:**

In the overall cohort, the all-cause mortality rates at 28 days, 90 days, and 1 year were 5.0%, 11.4%, and 22.3%, respectively. After adjusting for confounders, each 1-unit increase in BUN/Cr was linked to higher mortality at 28 days (HR = 1.039, 95% CI: 1.014–1.064), 90 days (HR = 1.038, 95% CI: 1.020–1.055), and 1 year (HR = 1.027, 95% CI: 1.013–1.041). A 1-standard deviation increase in BUN/Cr corresponded to a 45.0%, 43.6%, and 29.4% increased risk at each time point (all P < 0.05). Compared with the T1 group, patients in the T3 had significantly higher risks of 90-day (HR = 3.042, 95% CI: 1.742–5.312) and 1-year (HR = 1.971, 95% CI: 1.349–2.879) mortality. Subgroup analyses confirmed consistent associations between BUN/Cr and 90-day and 1-year mortality across various clinical subgroups. ROC curve analysis demonstrated that BUN/Cr had a moderate predictive ability for mortality at 28 days (AUC = 0.636), 90 days (AUC = 0.656), and 1 year (AUC = 0.610). Notably, adding BUN/Cr to the baseline multivariable model significantly improved discrimination for 28-day mortality (P for comparison = 0.012). RCS modeling showed a linear and positive association between BUN/Cr and mortality without significant non-linearity (P-nonlinear > 0.5 for all time points).

**Conclusion:**

A higher BUN/Cr ratio is significantly linked to increased short- and long-term mortality in SA-AKI patients aged 50 and above, suggesting its potential utility for early risk stratification and clinical decision-making.

## 1 Introduction

Sepsis is one of the most common and fatal clinical syndromes in intensive care medicine, often leading to systemic inflammatory response and multi-organ dysfunction (1). Acute kidney injury (AKI), a frequent complication of sepsis, is closely associated with poor prognosis (2). The incidence of AKI is significantly higher in sepsis patients, with studies showing that approximately 30% to 50% of sepsis patients develop acute kidney injury (2, 3). The occurrence of AKI not only exacerbates the clinical course of sepsis but also significantly increases the risk of patient mortality (4). Therefore, early identification of sepsis-associated AKI (SA-AKI) and its relationship with short-term and long-term mortality is crucial for improving patient outcomes.

Pathophysiologically, SA−AKI is a complex and heterogeneous syndrome that arises from the interplay of hemodynamic instability, systemic inflammation, and microvascular dysfunction, in which two major mechanistic pathways are commonly involved: a pre−renal mechanism driven by systemic hypotension, sepsis−induced myocardial depression, and intravascular volume depletion leading to impaired renal perfusion, and an intra−parenchymal mechanism characterized predominantly by ischemic and inflammatory injury to the renal tubular epithelium, with both pathways potentially coexisting and contributing to renal dysfunction to varying extents depending on the clinical context (5). Blood urea nitrogen (BUN) and creatinine (Cr) are commonly used biochemical markers for assessing renal function in clinical practice. Traditionally, the levels of BUN and Cr are used to evaluate kidney excretory function. However, recent studies have shown that the ratio of blood urea nitrogen to creatinine (BUN/Cr) may serve as a more valuable prognostic indicator for certain diseases (6–8). For example, Kang et al. found in a follow-up study of hospitalized patients with symptomatic heart failure (HF) from 2014 to 2016 that a high BUN/Cr ratio was associated with worse prognosis in both heart failure with reduced ejection fraction (HFrEF) and heart failure with preserved ejection fraction (HFpEF), showing increased short-term mortality in HFrEF and higher long-term all-cause mortality in HFpEF (6). Besides, Ma et al. found in a retrospective cohort study of 1,034 patients with trauma-related acute respiratory distress syndrome (ARDS) that a higher BUN/Cr ratio was significantly associated with an increased risk of in-hospital mortality, with this association remaining robust across multiple subgroups including age, sex, trauma severity, and physiological scores (7). In another retrospective cohort study using data from 850 critically ill patients with acute pancreatitis (AP), Wan et al. demonstrated that BUN/Cr ratio at intensive care unit (ICU) admission was a strong and independent predictor of both 28-day and 365-day mortality, with a J-shaped association showing a sharp rise in mortality risk when the ratio exceeded 16.80 (8). However, the value of BUN/Cr in assessing mortality risk in SA-AKI remains unknown. In addition, with the aging of the global population, the proportion of elderly patients with SA-AKI is gradually increasing (9). Compared with younger patients, elderly individuals with SA-AKI generally have poorer prognoses and higher mortality risks. Because of multiple comorbidities and the natural decline of kidney function with age, traditional renal function indicators may not accurately reflect the clinical outcomes of elderly patients. Therefore, conducting a focused analysis of patients aged 50 years and older to explore the relationship between the BUN/Cr ratio and both short- and long-term mortality could provide clinicians with more individualized prognostic assessment tools.

This study aims to explore the relationship between the BUN/Cr ratio and short- and long-term all-cause mortality in SA-AKI patients aged 50 and above through an analysis of a large cohort. This research will provide scientific evidence for early intervention and management of SA-AKI patients, ultimately helping to improve clinical outcomes.

## 2 Methods

### 2.1 Study population

This single-center, retrospective cohort study utilized patient data from Henan Provincial People’s Hospital between January 2020 and August 2024. The study included all patients who were hospitalized with SA-AKI. After applying the following inclusion and exclusion criteria, a total of 764 patients were eligible for the study.

Inclusion criteria: (1) Patients aged 50 years or older; (2) Meeting the diagnostic criteria for SA-AKI based on the Acute Disease Quality Initiative (ADQI) 2023 definition, in which AKI occurs within 7 days following the onset of sepsis (10); (3) Patients who did not have end-stage kidney disease (ESKD) at the time of admission or during hospitalization and were not undergoing dialysis; (4) Patients with complete clinical data, including BUN, serum Cr, and other relevant biochemical markers. Exclusion criteria: (1) Patients with end-stage liver disease, severe hematologic disorders, or immune system diseases; (2) Patients with AKI prior to hospital admission; (3) Patients who died or were transferred within 24 hours of admission, thus failing to complete the full course of hospitalization; (4) Patients lost to follow-up.

The clinical data and study protocol were approved by the Ethics Committee of Henan Provincial People’s Hospital. As a retrospective cohort study using anonymized electronic medical records, the requirement for informed consent was waived by the ethics committee. The study was conducted in accordance with the ethical principles of the Declaration of Helsinki.

### 2.2 Data collection and definition

All clinical data used in this study were extracted from the electronic medical record system of Henan Provincial People’s Hospital. Two independent investigators carried out the data extraction separately and cross-checked the results to enhance consistency and accuracy. Collected variables included demographic characteristics (age and sex), comorbidities, vital signs, laboratory findings, organ dysfunction scoring systems, major interventions, and prognostic outcomes.

For comorbidities, diabetes was identified based on a prior diagnosis or the use of antihyperglycemic medications during hospitalization. Heart failure was defined by evidence of cardiac dysfunction on echocardiography or the presence of typical clinical symptoms and signs (11). Myocardial infarction required a combination of classic symptoms, electrocardiographic changes, and elevated cardiac biomarkers, or a documented prior diagnosis (12). Cerebrovascular diseases included a history of ischemic stroke, hemorrhagic stroke, or transient ischemic attack (TIA). Malignancy referred to any confirmed solid or hematologic tumor, whether active or under treatment during the hospital stay. ARDS was diagnosed based on the 2012 Berlin Definition, which includes acute onset, bilateral pulmonary infiltrates, a ratio of partial pressure of arterial oxygen to fraction of inspired oxygen (PaO_2_/FiO_2_) less than 300 mmHg, and exclusion of cardiogenic causes (13).

Organ dysfunction and disease severity were assessed using the Sequential Organ Failure Assessment (SOFA) score, Simplified Acute Physiology Score II (SAPS II), Oxford Acute Severity of Illness Score (OASIS), and the Glasgow Coma Scale (GCS). The most severe values within 24 hours following the diagnosis of sepsis were recorded for all scores. AKI was staged according to the 2012 Kidney Disease: Improving Global Outcomes (KDIGO) criteria, incorporating both serum creatinine and urine output, with the highest observed stage during hospitalization recorded as the final classification (14).

Vital signs were collected within 24 hours after sepsis diagnosis. Maximum values were recorded for body temperature, heart rate, and respiratory rate, while the minimum values were used for systolic blood pressure (SBP), diastolic blood pressure (DBP), mean arterial pressure (MAP), and oxygen saturation (SpO_2_). Arterial blood gas parameters—including pH, partial pressure of oxygen (PaO_2_), partial pressure of carbon dioxide (PaCO_2_), lactate, arterial oxygen saturation (SaO_2_), and fraction of inspired oxygen (FiO_2_)—were collected within the first 24 hours following sepsis diagnosis, with minimum values used for pH, PaO_2_, and SaO_2_ to reflect the lowest acid-base balance and oxygenation levels, and maximum values used for PaCO_2_, lactate, and FiO_2_ to capture the most severe respiratory compromise, metabolic stress, and oxygen supplementation requirements. Additionally, the PO_2_/FiO_2_ ratio was calculated based on these values to evaluate the severity of hypoxemia and oxygenation impairment.

Laboratory tests performed within the first 24 hours following the diagnosis of sepsis encompassed complete blood count (CBC), electrolyte panels, liver and kidney function markers, and coagulation profiles. Specifically, the highest recorded value was used for white blood cell (WBC) count, while lowest values were selected for hemoglobin and platelet count. For electrolytes and metabolic indices, minimum values were used for sodium and chloride, and maximum values for potassium, calcium, magnesium, and phosphate. Liver function parameters—including alanine aminotransferase (ALT), aspartate aminotransferase (AST), and total bilirubin—were assessed using their peak values, whereas lowest levels were recorded for albumin. Kidney function was evaluated based on the maximum levels of blood urea nitrogen (BUN), serum Cr and estimated glomerular filtration rate (eGFR). Coagulation tests included maximum values for prothrombin time (PT), activated partial thromboplastin time (APTT), and fibrinogen. Urine output was quantified as the total volume (mL) collected within the first 24 hours after admission to ICU. As for treatment measures, the use of mechanical ventilation and renal replacement therapy (RRT) within 48 hours of ICU entry was documented. In addition, for vasoactive medications administered within the first 48 hours of ICU admission, the maximum total dose, peak infusion rate, and duration of use were recorded for norepinephrine, epinephrine, dopamine, and dobutamine.

### 2.3 Definition and classification of the BUN/Cr ratio

In this study, BUN/Cr was used as a key exposure variable to assess renal function and its association with clinical outcomes. Both BUN and serum Cr were measured in mg/dL, and values were obtained from the first laboratory tests within 24 hours following the diagnosis of sepsis. The BUN/Cr ratio was calculated by directly dividing the BUN value by the Cr value. For statistical modeling, the BUN/Cr ratio was analyzed both as a continuous variable and in standardized form. Standardization was performed using the Z-score method, calculated as (BUN/Cr – mean) divided by the standard deviation (SD), to minimize scale-related effects and allow for comparison of effect sizes across models. The standardized ratio was included in multivariable regression analyses to assess the risk associated with each one SD increase in BUN/Cr.

To further investigate the potential non-linear association between BUN/Cr and clinical outcomes, patients were also divided into three groups according to tertiles of the BUN/Cr distribution: T1 (≤ 16.00, n = 256), T2 (16.01–22.50, n = 254), and T3 (> 22.50, n = 254).

### 2.4 Definition of all-cause mortality

The primary outcome of this study was post-discharge all-cause mortality, assessed at three specific time points: 28 days, 90 days, and 1 year after discharge. All-cause mortality was defined as death due to any cause during the follow-up period, regardless of whether it was directly related to sepsis, AKI, or any underlying condition. Mortality data were primarily obtained from historical hospitalization records within the hospital’s electronic medical record system. For patients with missing or incomplete follow-up data, additional confirmation was conducted through telephone follow-ups by the research team. The date of death was used to determine whether it occurred within the specified time windows, allowing for the calculation of 28-day, 90-day, and 1-year mortality rates.

### 2.5 Statistical methods

All statistical analyses were conducted using R software (version 4.4.3, R Foundation for Statistical Computing, Vienna, Austria). The Shapiro-Wilk test was first applied to continuous variables to evaluate their distribution. Variables not conforming to a normal distribution were summarized using medians and interquartile ranges (IQR), and group comparisons were performed using the Kruskal-Wallis rank-sum test. Categorical variables were presented as counts and percentages, with group differences assessed by chi-square test or Fisher’s exact test as appropriate. Patients were divided into tertiles based on their BUN/Cr: T1 (≤ 16.0), T2 (16.01–22.50), and T3 (> 22.50). These groupings were used to explore differences in baseline characteristics, clinical interventions, and outcome indicators.

To investigate the association between BUN/Cr and post-discharge all-cause mortality at 28 days, 90 days, and 1 year, multivariable Cox proportional hazards regression models were constructed. Three hierarchical models were developed for each time point. Model 1 was an unadjusted univariable model. Model 2 adjusted for age. Model 3 included additional covariates tailored to each endpoint: for 28-day mortality, Model 3 adjusted for age, cerebrovascular disease, ARDS, and PaO_2_; for 90-day mortality, adjustments included cerebrovascular disease, ARDS, malignancy, SBP, PaO_2_, FiO_2_, hemoglobin, and duration of norepinephrine use; for 1-year mortality, further adjustments included ICU length of stay, cerebrovascular disease, ARDS, malignancy, PaO_2_, FiO_2_, platelet count, hemoglobin, AST, ALT, GCS score, SAPS II score, OASIS score, maximum total dose of epinephrine, and duration of epinephrine use. Subgroup analyses were also performed to examine the consistency of associations between BUN/Cr and mortality across different patient strata, including age, gender, AKI stage, presence of ARDS, heart failure, and diabetes. To assess the predictive performance of BUN/Cr at each mortality time point, receiver operating characteristic (ROC) curves were generated, and the area under the curve (AUC) was calculated. In addition, to evaluate the incremental prognostic value of BUN/Cr beyond established clinical predictors, a baseline prediction model was constructed including all covariates adjusted in Model 3 for each respective endpoint. Subsequently, BUN/Cr was incorporated into the baseline model, and the change in discriminative performance was assessed by comparing AUC values using the DeLong test. Additionally, restricted cubic spline (RCS) models were applied to examine potential non-linear dose-response relationships between BUN/Cr and the risk of mortality. All statistical tests were two-sided, and a P-value less than 0.05 was considered statistically significant.

## 3 Results

### 3.1 The baseline characteristics grouped by the BUN/Cr tertiles

After dividing the 764 patients into tertiles based on their BUN/Cr (T1: ≤ 16.0; T2: 16.01–22.50; T3: > 22.50), baseline characteristics, laboratory values, clinical interventions, and outcomes were compared across groups (**Table 1**). Several variables demonstrated statistically significant differences. Among the positive findings, gender distribution differed significantly, with the highest proportion of males in the T1 group (67.2%) and the lowest in T3 (55.1%, P = 0.016). The prevalence of cerebrovascular disease was highest in T2 (17.7%, P = 0.031). AKI staging also varied across groups, with T1 showing the highest proportion of stage III AKI (43.4%, P = 0.019). The need for RRT decreased progressively with rising BUN/Cr levels (T1: 17.6% *vs*. T3: 6.3%, P < 0.001). In terms of vital signs, T1 patients had the highest respiratory and heart rates (P = 0.009 and 0.041, respectively), while DBP was lowest in T3 (P = 0.038). For arterial blood gas parameters, pH was lowest in T1, PaO_2_ peaked in T2 and was lowest in T3 (all P < 0.001), and FiO_2_ differed significantly across groups (P = 0.012). The PO_2_/FiO_2_ ratio also showed statistically significant variation among the BUN/Cr tertiles (P = 0.008), with the highest median value observed in T2 and the lowest in T3. In terms of renal function markers, serum creatinine levels showed a modest but significant difference across groups (P < 0.001), while the eGFR revealed more pronounced variation (P < 0.001), with the lowest median eGFR in T1 and the highest in T2. Electrolytes including chloride, magnesium, calcium, and phosphate showed significant variation (P < 0.05), with lactate levels being highest in T1 (P < 0.001). Regarding laboratory findings, hemoglobin levels were significantly lower in the T3 group (P < 0.001), while AST and ALT were markedly higher in T1 (P = 0.002 and 0.031). Prothrombin time was longer in T3 (P = 0.006). Among severity scores, the T1 group had higher SOFA and OASIS scores and lower GCS scores compared to other groups (all P < 0.05). For prognosis, the T3 group had significantly higher 90-day and 1-year all-cause mortality rates compared with T1 and T2 (P < 0.001).

**Table 1:** The baseline characteristics grouped by the BUN/Cr tertiles.

Variables	Overall	T1	T2	T3	P value
N	764	256	254	254	
Age	65.35 (58.39, 74.85)	64.07 (57.59, 72.32)	65.78 (58.48, 76.21)	66.20 (59.04, 75.62)	0.095
Gender					0.016
Male	473 (61.9%)	172 (67.2%)	161 (63.4%)	140 (55.1%)	
Female	291 (38.1%)	84 (32.8%)	93 (36.6%)	114 (44.9%)	
Diabetes	266 (34.8%)	86 (33.6%)	89 (35.0%)	91 (35.8%)	0.866
Heart failure	209 (27.4%)	63 (24.6%)	67 (26.4%)	79 (31.1%)	0.236
Myocardial infarction	139 (18.2%)	50 (19.5%)	50 (19.7%)	39 (15.4%)	0.356
Cerebrovascular diseases	101 (13.2%)	30 (11.7%)	45 (17.7%)	26 (10.2%)	0.031
ARDS	450 (58.9%)	155 (60.5%)	139 (54.7%)	156 (61.4%)	0.249
Malignancy	127 (16.6%)	41 (16.0%)	44 (17.3%)	42 (16.5%)	0.923
AKI staging					0.019
I	137 (17.9%)	40 (15.6%)	50 (19.7%)	47 (18.5%)	
II	358 (46.9%)	105 (41.0%)	129 (50.8%)	124 (48.8%)	
III	269 (35.2%)	111 (43.4%)	75 (29.5%)	83 (32.7%)	
Mechanical ventilation_48h_	707 (92.5%)	239 (93.4%)	240 (94.5%)	228 (89.8%)	0.106
Renal replacement therapy_48h_	85 (11.1%)	45 (17.6%)	24 (9.4%)	16 (6.3%)	< 0.001
ICU length of stay	6.55 (3.69, 11.65)	7.04 (4.24, 11.78)	5.93 (3.17, 10.68)	6.58 (3.73, 12.13)	0.059
Temperature_max_	37.44 (37.06, 38.06)	37.50 (37.06, 38.06)	37.44 (37.11, 38.10)	37.39 (37.06, 38.00)	0.580
Heart rate_max_	108.00 (94.00, 123.00)	109.00 (96.00, 123.50)	104.00 (90.00, 122.00)	109.00 (96.00, 123.00)	0.041
Respiratory rate_max_	27.50 (24.00, 31.00)	28.00 (24.00, 32.75)	27.00 (23.00, 30.00)	27.00 (24.00, 31.00)	0.009
SBP_min_	84.00 (75.00, 92.00)	83.00 (73.00, 90.00)	84.00 (76.00, 92.00)	85.00 (75.00, 93.00)	0.305
DBP_min_	44.00 (39.00, 49.00)	44.00 (40.00, 49.00)	44.00 (39.00, 50.00)	43.00 (38.00, 48.00)	0.038
MAP_min_	57.00 (50.00, 63.00)	56.00 (50.00, 62.50)	58.00 (49.00, 64.00)	56.00 (50.00, 62.00)	0.308
Oxygen saturation_min_	93.00 (90.50, 96.00)	93.00 (90.00, 95.00)	94.00 (91.00, 96.00)	93.00 (90.00, 96.00)	0.171
ICU Urine output_24h_	1,288.50 (762.00, 2,007.50)	1,2.00.00 (579.00, 2,018.00)	1,260.00 (800.00, 1,990.00)	1,360.00 (803.00, 2,027.00)	0.071
Arterial oxygen saturation_min_	84.78 (81.00, 96.00)	84.78 (78.00, 96.00)	84.78 (82.00, 97.00)	84.78 (83.00, 95.00)	0.441
PH_min_	7.34 (7.24, 7.40)	7.30 (7.21, 7.38)	7.35 (7.27, 7.41)	7.35 (7.27, 7.41)	< 0.001
PO_2min_	110.00 (59.00, 230.00)	98.50 (57.00, 238.50)	134.50 (77.00, 283.00)	93.50 (51.00, 178.00)	< 0.001
PCO_2max_	41.00 (35.00, 48.00)	42.00 (35.00, 48.50)	40.00 (36.00, 46.00)	41.00 (34.00, 49.00)	0.539
FiO_2max_	60.00 (50.00, 100.00)	60.00 (50.00, 100.00)	70.00 (50.00, 100.00)	60.00 (50.00, 100.00)	0.012
PO_2min_/FiO_2max_	1.76 (0.88, 3.42)	1.71 (0.82, 3.32)	2.16 (1.07, 3.88)	1.58 (0.88, 3.02)	0.008
Potassium_max_	4.20 (3.80, 4.80)	4.30 (3.80, 5.00)	4.20 (3.80, 4.70)	4.20 (3.70, 4.90)	0.058
Sodium_min_	139.00 (136.00, 142.00)	138.00 (135.00, 141.00)	139.00 (136.00, 142.00)	139.00 (136.00, 142.00)	0.142
Chloride_min_	104.00 (100.00, 108.00)	103.00 (99.00, 107.00)	104.00 (101.00, 108.00)	104.00 (99.00, 109.00)	0.025
Magnesium_max_	1.90 (1.60, 2.20)	1.80 (1.55, 2.10)	1.90 (1.60, 2.10)	2.00 (1.70, 2.20)	< 0.001
Calcium_max_	8.10 (7.50, 8.80)	8.00 (7.30, 8.70)	8.20 (7.50, 8.80)	8.10 (7.60, 8.80)	0.036
Phosphate_max_	4.00 (3.10, 5.20)	4.30 (3.15, 5.90)	3.90 (3.10, 4.90)	3.80 (3.00, 4.80)	< 0.001
Lactate_max_	2.10 (1.40, 3.60)	2.50 (1.40, 4.70)	2.00 (1.40, 3.20)	1.90 (1.40, 2.90)	< 0.001
White blood cell count_max_	11.05 (7.50, 16.85)	12.10 (8.30, 17.00)	10.60 (6.70, 16.50)	10.95 (7.30, 15.90)	0.077
Platelets_min_	171.00 (107.00, 243.00)	171.00 (109.50, 239.50)	176.00 (111.00, 243.00)	166.50 (102.00, 244.00)	0.786
Hemoglobin_min_	10.93 ± 2.67	11.12 ± 2.56	11.27 ± 2.56	10.41 ± 2.81	< 0.001
Blood urea nitrogen_max_	24.00 (16.00, 40.50)	17.50 (13.00, 33.00)	20.00 (15.00, 33.00)	32.00 (23.00, 49.00)	< 0.001
Creatinine_max_	1.10 (0.90, 1.80)	1.40 (1.00, 3.05)	1.10 (0.90, 1.60)	1.00 (0.80, 1.60)	< 0.001
eGFR_min_	65.18 (34.82, 93.58)	51.71 (19.32, 82.01)	72.85 (39.24, 97.84)	71.14 (41.27, 102.02)	< 0.001
Total bilirubin_max_	0.90 (0.50, 2.10)	0.90 (0.40, 1.95)	0.80 (0.50, 2.00)	0.95 (0.50, 2.40)	0.457
Aspartate aminotransferase_max_	63.00 (31.00, 198.00)	85.50 (37.00, 282.50)	53.00 (27.00, 188.00)	55.00 (29.00, 141.00)	0.002
Alanine aminotransferase_max_	36.50 (20.00, 113.50)	44.00 (22.00, 141.00)	32.00 (19.00, 113.00)	33.50 (19.00, 85.00)	0.031
Albumin_min_	3.00 (2.60, 3.45)	3.00 (2.60, 3.50)	3.00 (2.60, 3.50)	2.90 (2.50, 3.40)	0.157
Prothrombin time_max_	15.00 (12.70, 19.50)	14.45 (12.60, 18.10)	14.75 (12.40, 18.30)	15.50 (13.20, 20.60)	0.006
APTT_max_	32.40 (27.80, 43.45)	32.90 (27.75, 44.55)	32.30 (28.00, 39.70)	32.35 (27.70, 45.00)	0.722
Fibrinogen_max_	269.00 (179.50, 417.00)	284.00 (190.00, 406.00)	252.00 (170.00, 398.00)	276.00 (180.00, 445.00)	0.101
SOFA score_24h_	9.00 (6.00, 11.00)	10.00 (7.00, 12.00)	8.00 (6.00, 11.00)	8.00 (6.00, 11.00)	< 0.001
GCS score_24h_	5.00 (2.00, 8.00)	4.00 (2.00, 7.00)	5.00 (2.00, 8.00)	6.00 (2.00, 8.00)	0.010
SAPS II score_24h_	45.00 (37.00, 55.00)	48.00 (36.50, 57.50)	43.00 (36.00, 55.00)	44.00 (38.00, 54.00)	0.106
OASIS score_24h_	39.00 (34.00, 44.00)	40.00 (35.00, 46.00)	38.00 (33.00, 43.00)	38.00 (34.00, 43.00)	0.013
All-cause mortality_28-day_	38 (5.0%)	9 (3.5%)	10 (3.9%)	19 (7.5%)	0.078
All-cause mortality_90-day_	87 (11.4%)	17 (6.6%)	22 (8.7%)	48 (18.9%)	< 0.001
All-cause mortality_1-year_	170 (22.3%)	43 (16.8%)	48 (18.9%)	79 (31.1%)	< 0.001

BUN, blood urea nitrogen; Cr, creatinine; ARDS, acute respiratory distress syndrome; AKI, acute kidney injury; ICU, intensive care unit; SBP, systolic blood pressure; DBP, diastolic blood pressure; MAP, mean arterial pressure; PO_2_, partial pressure of oxygen; PCO_2_, partial pressure of carbon dioxide; FiO_2_, fraction of inspired oxygen; AST, aspartate aminotransferase; ALT, alanine aminotransferase; APTT, activated partial thromboplastin time; SOFA, Sequential Organ Failure Assessment; GCS, Glasgow Coma Scale; SAPS II, Simplified Acute Physiology Score II; OASIS, Oxford Acute Severity of Illness Score.

Conversely, no statistically significant differences were noted for several variables, including age, diabetes, heart failure, myocardial infarction, ARDS, malignancy, use of mechanical ventilation, ICU length of stay, body temperature, SBP, MAP, oxygen saturation, urine output, arterial oxygen saturation, PCO_2_, potassium, sodium, WBC and platelet counts, total bilirubin, albumin, APTT, fibrinogen, SAPS II score, and 28-day mortality (all P > 0.05).

### 3.2 Association between BUN/Cr and post-discharge mortality

In the Cox proportional hazards analysis (**Table 2**), BUN/Cr was significantly associated with post-discharge all-cause mortality across 28-day, 90-day, and 1-year timeframes. When considered as a continuous variable, each 1-unit increase in BUN/Cr was linked to significantly higher mortality risk in the unadjusted model (Model 1): 28-day (HR = 1.039, P = 0.002), 90-day (HR = 1.039, P < 0.001), and 1-year (HR = 1.028, P < 0.001). After standardization (per 1-SD increase), mortality risk rose by 45.4% (HR = 1.454, P = 0.002), 45.9% (HR = 1.459, P < 0.001), and 31.3% (HR = 1.313, P < 0.001) for 28-day, 90-day, and 1-year mortality, respectively.

**Table 2:** Association between BUN/Cr and mortality: multivariate Cox regression analysis.

Variables	Model 1			Model 2			Model 3		
	HR	95% CI	P value	HR	95% CI	P value	HR	95% CI	P value
28-day mortality									
Categorical variable									
T1 (≤ 16.0)	Ref								
T2 (16.0-22.5)	1.121	0.455-2.758	0.804	1.073	0.435-2.643	0.879	1.093	0.441-2.710	0.848
T3 (> 22.5)	2.175	0.984-4.807	0.055	2.077	0.939-4.596	0.071	2.074	0.936-4.594	0.072
P for trend			0.084			0.101			0.112
Continuous variable									
BUN/Cr	1.039	1.014-1.064	0.002	1.038	1.013-1.064	0.003	1.039	1.014-1.064	0.002
Standardized BUN/Cr	1.454	1.146-1.844	0.002	1.446	1.136-1.841	0.003	1.450	1.146-1.835	0.002
90-day mortality									
Categorical variable									
T1 (≤ 16.0)	Ref								
T2 (16.0-22.5)	1.311	0.696-2.469	0.402	1.263	0.670-2.379	0.471	1.279	0.677-2.418	0.448
T3 (> 22.5)	3.014	1.733-5.241	< 0.001	2.904	1.669-5.052	< 0.001	3.042	1.742-5.312	< 0.001
P for trend			< 0.001			< 0.001			< 0.001
Continuous variable									
BUN/Cr	1.039	1.023-1.056	< 0.001	1.039	1.022-1.056	< 0.001	1.038	1.020-1.055	< 0.001
Standardized BUN/Cr	1.459	1.245-1.710	< 0.001	1.452	1.237-1.706	< 0.001	1.436	1.219-1.692	< 0.001
1-year mortality									
Categorical variable									
T1 (≤ 16.0)	Ref								
T2 (16.0-22.5)	1.140	0.756-1.721	0.532	1.081	0.716-1.633	0.709	1.117	0.737-1.694	0.601
T3 (> 22.5)	2.073	1.430-3.006	< 0.001	1.979	1.364-2.870	< 0.001	1.971	1.349-2.879	< 0.001
P for trend			< 0.001			< 0.001			< 0.001
Continuous variable									
BUN/Cr	1.028	1.015-1.041	< 0.001	1.027	1.014-1.041	< 0.001	1.027	1.013-1.041	< 0.001
Standardized BUN/Cr	1.313	1.160-1.487	<0.001	1.301	1.147-1.477	< 0.001	1.294	1.134-1.477	< 0.001

For 28-day mortality: Model 1 univariate; Model 2 adjusted for age; Model 3 adjusted for age, cerebrovascular disease, ARDS, and PO_2_.

For 90-day mortality: Model 1 univariate; Model 2 adjusted for age; Model 3 adjusted for age, cerebrovascular disease, ARDS, malignancy, SBP, PO_2_, FiO_2_, hemoglobin, and duration of norepinephrine use.

For 1-year mortality: Model 1 univariate; Model 2 adjusted for age; Model 3 adjusted for age, ICU length of stay, cerebrovascular disease, ARDS, malignancy, PO_2_, FiO_2_, platelet count, hemoglobin, AST, ALT, GCS 24h_min_, SAPS II score, OASIS score, maximum total dose of epinephrine, and duration of epinephrine use.

BUN, blood urea nitrogen; Cr, creatinine; HR, hazard ratio; CI, confidence interval; ARDS, acute respiratory distress syndrome; PO_2_, partial pressure of oxygen; SBP, systolic blood pressure; FiO_2_, fraction of inspired oxygen; ICU, intensive care unit; AST, aspartate aminotransferase; ALT, alanine aminotransferase; GCS, Glasgow Coma Scale; SAPS II, Simplified Acute Physiology Score II; OASIS, Oxford Acute Severity of Illness Score.

In Model 2, which adjusted only for age, the associations persisted: each 1-unit increase in BUN/Cr was associated with a 3.8% higher risk of 28-day mortality (HR = 1.038, P = 0.003), 3.9% for 90-day (HR = 1.039, P < 0.001), and 2.7% for 1-year mortality (HR = 1.027, P < 0.001). The standardized BUN/Cr remained predictive: 44.6% increase for 28-day (HR = 1.446, P = 0.003), 45.2% for 90-day (HR = 1.452, P < 0.001), and 30.1% for 1-year mortality (HR = 1.301, P < 0.001).

In the fully adjusted Model 3, the associations remained statistically significant. Each unit increase in BUN/Cr corresponded to a higher risk of death: 28-day (HR = 1.039, 95% CI: 1.014–1.064, P = 0.002), 90-day (HR = 1.038, 95% CI: 1.020–1.055, P < 0.001), and 1-year (HR = 1.027, 95% CI: 1.013–1.041, P < 0.001). Standardized BUN/Cr remained an independent predictor with risk increases of 45.0% (HR = 1.450, 95% CI: 1.146–1.835, P = 0.002), 43.6% (HR = 1.436, 95% CI: 1.219–1.692, P < 0.001), and 29.4% (HR = 1.294, 95% CI: 1.134–1.477, P < 0.001), respectively.

When BUN/Cr was treated as a categorical variable, using T1 (≤ 16.0) as reference, T3 (> 22.5) showed a consistent trend toward increased 28-day mortality: Model 1 (HR = 2.175, P = 0.055), Model 2 (HR = 2.077, P = 0.071), and Model 3 (HR = 2.074, P = 0.072), although none reached statistical significance. In contrast, for 90-day mortality, T3 was significantly associated with increased risk across all models: Model 1 (HR = 3.014, P < 0.001), Model 2 (HR = 2.904, P < 0.001), and Model 3 (HR = 3.042, 95% CI: 1.742–5.312, P < 0.001). For 1-year mortality, the pattern was similar: the T3 group showed significantly elevated risk in Model 1 (HR = 2.073, P < 0.001), Model 2 (HR = 1.979, P < 0.001), and Model 3 (HR = 1.971, 95% CI: 1.349–2.879, P < 0.001).

### 3.3 Subgroup analysis of BUN/Cr and 90-day mortality

Subgroup analyses (**Table 3**) explored the consistency of the association between BUN/Cr and 90-day all-cause mortality across various patient strata. Among patients aged ≤ 65 years, each 1-unit increase in BUN/Cr was associated with a significantly higher mortality risk (HR = 1.039, P = 0.008), and a 1-SD increase in standardized BUN/Cr corresponded to a 46.0% increased risk (HR = 1.460, P = 0.008). In contrast, patients over 65 years had a stronger association: BUN/Cr (HR = 1.033, P = 0.003), standardized BUN/Cr (HR = 1.381, P = 0.003), and T3 *vs*. T1 (HR = 3.962, P = 0.001) were all significant.

**Table 3:** Subgroup analysis of the association between BUN/Cr and 90-day mortality.

Subgroups	T2 *vs*. T1		T3 *vs*. T1		BUN/Cr		Standardized BUN/Cr	
	HR (95% CI)	P value	HR (95% CI)	P value	HR (95% CI)	P value	HR (95% CI)	P value
Age								
≤ 65 years	0.907 (0.360, 2.285)	0.836	1.912 (0.851, 4.299)	0.117	1.039 (1.010, 1.069)	0.008	1.460 (1.104, 1.931)	0.008
> 65 years	1.684 (0.668, 4.243)	0.269	3.962 (1.742, 9.012)	0.001	1.033 (1.011, 1.056)	0.003	1.381 (1.117, 1.707)	0.003
Gender								
Male	1.001 (0.481, 2.084)	0.998	2.049 (1.070, 3.923)	0.030	1.034 (1.011, 1.058)	0.004	1.385 (1.108, 1.732)	0.004
Female	3.866 (0.820, 18.224)	0.087	8.091 (1.887, 34.681)	0.005	1.040 (1.014, 1.068)	0.003	1.475 (1.145, 1.900)	0.003
AKI staging								
Stage 1	0.482 (0.059, 3.939)	0.496	1.540 (0.335, 7.075)	0.579	1.048 (1.000, 1.098)	0.048	1.587 (1.003, 2.511)	0.048
Stage 2	1.442 (0.500, 4.161)	0.498	3.552 (1.336, 9.443)	0.011	1.039 (1.012, 1.067)	0.005	1.456 (1.120, 1.893)	0.005
Stage 3	1.443 (0.572, 3.640)	0.437	3.759 (1.700, 8.308)	0.001	1.037 (1.014, 1.061)	0.002	1.427 (1.143, 1.781)	0.002
ARDS								
Yes	1.444 (0.700, 2.983)	0.320	3.020 (1.590, 5.736)	0.001	1.035 (1.015, 1.055)	0.001	1.402 (1.157, 1.698)	0.001
No	1.201 (0.299, 4.826)	0.796	3.194 (1.040, 9.807)	0.042	1.052 (1.021, 1.084)	0.001	1.642 (1.221, 2.209)	0.001
Heart failure								
Yes	1.362 (0.304, 6.103)	0.687	3.799 (1.095, 13.178)	0.035	1.031 (0.989, 1.075)	0.147	1.350 (0.900, 2.024)	0.147
No	1.426 (0.705, 2.884)	0.323	2.645 (1.406, 4.977)	0.003	1.035 (1.016, 1.055)	< 0.001	1.401 (1.164, 1.686)	< 0.001
Diabetes								
Yes	1.062 (0.375, 3.011)	0.910	2.412 (1.004, 5.798)	0.049	1.043 (1.012, 1.075)	0.006	1.510 (1.124, 2.028)	0.006
No	1.450 (0.641, 3.280)	0.372	3.249 (1.576, 6.696)	0.001	1.040 (1.018, 1.061)	< 0.001	1.463 (1.194, 1.793)	< 0.001

Subgroup analysis adjusted for age, cerebrovascular disease, ARDS, malignancy, SBP, PO_2_, FiO_2_, hemoglobin, and duration of norepinephrine use.

BUN, blood urea nitrogen; Cr, creatinine; HR, hazard ratio; CI, confidence interval; AKI, acute kidney injury; ARDS, acute respiratory distress syndrome; SBP, systolic blood pressure; PO_2_, partial pressure of oxygen; FiO_2_, fraction of inspired oxygen.

In gender-based analysis, both males and females showed significant associations with continuous BUN/Cr (male: HR = 1.034, P = 0.004; female: HR = 1.040, P = 0.003) and standardized BUN/Cr (male: HR = 1.385, P = 0.004; female: HR = 1.475, P = 0.003). However, T3 *vs*. T1 was only significant in females (HR = 8.091, P = 0.005), while in males it remained marginal (HR = 2.049, P = 0.030).

Across AKI stages, the positive association remained. For stage 1, the trend was marginally significant for BUN/Cr (HR = 1.048, P = 0.048) and standardized BUN/Cr (HR = 1.587, P = 0.048). In stage 2, all indicators reached significance, with T3 *vs*. T1 (HR = 3.552, P = 0.011), BUN/Cr (HR = 1.039, P = 0.005), and standardized BUN/Cr (HR = 1.456, P = 0.005). Similarly, in stage 3, T3 *vs*. T1 showed a significantly elevated risk (HR = 3.759, P = 0.001), along with BUN/Cr (HR = 1.037, P = 0.002) and standardized BUN/Cr (HR = 1.427, P = 0.002).

In patients with ARDS, T3 *vs*. T1 (HR = 3.020, P = 0.001), BUN/Cr (HR = 1.035, P = 0.001), and standardized BUN/Cr (HR = 1.402, P = 0.001) were significantly associated with increased mortality risk. Similar trends were seen in the non-ARDS group, particularly for T3 *vs*. T1 (HR = 3.194, P = 0.042), BUN/Cr (HR = 1.052, P = 0.001), and standardized BUN/Cr (HR = 1.642, P = 0.001).

Among those with heart failure, only T3 *vs*. T1 reached significance (HR = 3.799, P = 0.035). In contrast, for patients without heart failure, all indicators were significant: T3 *vs*. T1 (HR = 2.645, P = 0.003), BUN/Cr (HR = 1.035, P < 0.001), and standardized BUN/Cr (HR = 1.401, P < 0.001). Finally, in the diabetes subgroup, T3 *vs*. T1 reached significance (HR = 2.412, P = 0.049), as did BUN/Cr (HR = 1.043, P = 0.006) and standardized BUN/Cr (HR = 1.510, P = 0.006). In non-diabetic patients, the associations remained consistent and robust across all metrics: T3 *vs*. T1 (HR = 3.249, P = 0.001), BUN/Cr (HR = 1.040, P < 0.001), and standardized BUN/Cr (HR = 1.463, P < 0.001).

Overall, while the association between BUN/Cr and 90-day mortality remained statistically significant in most subgroups—particularly when analyzed as a continuous or standardized variable—the strength and statistical significance of the association were not uniform across all strata. In several subgroups, especially when using tertile-based comparisons (T2 *vs*. T1), the risk estimates did not reach statistical significance, suggesting that BUN/Cr may not serve as an equally strong discriminator in every clinical context. These findings indicate a degree of heterogeneity in predictive performance across patient subgroups.

### 3.4 Subgroup analysis of BUN/Cr and 1-year mortality

Subgroup analysis for 1-year all-cause mortality (**Table 4**) further examined the relationship between BUN/Cr and patient outcomes across stratified clinical characteristics. In patients aged > 65 years, BUN/Cr was significantly associated with increased mortality—both as a continuous variable (HR = 1.029, P = 0.001) and per 1 SD (HR = 1.320, P = 0.001)—and patients in T3 had significantly higher risk compared to T1 (HR = 2.137, P = 0.002).

**Table 4:** Subgroup analysis of the association between BUN/Cr and 1-year mortality.

Subgroups	T2 *vs*. T1		T3 *vs*. T1		BUN/Cr		Standardized BUN/Cr	
	HR (95% CI)	P value	HR (95% CI)	P value	HR (95% CI)	P value	HR (95% CI)	P value
Age								
≤ 65 years	0.900 (0.458, 1.769)	0.760	1.354 (0.727, 2.521)	0.339	1.017 (0.991, 1.043)	0.199	1.176 (0.918, 1.506)	0.199
> 65 years	1.212 (0.699, 2.100)	0.493	2.137 (1.309, 3.488)	0.002	1.029 (1.012, 1.046)	0.001	1.320 (1.126, 1.548)	0.001
Gender								
Male	1.045 (0.631, 1.729)	0.865	1.679 (1.056, 2.668)	0.029	1.024 (1.006, 1.042)	0.010	1.256 (1.057, 1.493)	0.010
Female	3.866 (0.820, 18.224)	0.087	8.091 (1.887, 34.681)	0.005	1.040 (1.014, 1.068)	0.003	1.357 (1.102, 1.671)	0.004
AKI staging								
Stage 1	1.220 (0.304, 4.902)	0.779	1.831 (0.554, 6.051)	0.321	1.045 (0.997, 1.095)	0.065	1.538 (0.973, 2.429)	0.065
Stage 2	0.875 (0.478, 1.602)	0.665	1.680 (0.964, 2.926)	0.067	1.021 (1.001, 1.042)	0.038	1.230 (1.012, 1.495)	0.038
Stage 3	1.373 (0.698, 2.703)	0.359	2.418 (1.314, 4.450)	0.005	1.043 (1.019, 1.067)	< 0.001	1.508 (1.207, 1.883)	< 0.001
ARDS								
Yes	1.303 (0.801, 2.120)	0.287	1.871 (1.198, 2.922)	0.006	1.024 (1.008, 1.041)	0.003	1.268 (1.082, 1.485)	0.003
No	0.973 (0.423, 2.236)	0.948	2.034 (0.959, 4.316)	0.064	1.030 (1.006, 1.054)	0.015	1.333 (1.056, 1.683)	0.015
Heart failure								
Yes	1.560 (0.656, 3.710)	0.315	2.135 (0.990, 4.607)	0.053	1.017 (0.990, 1.046)	0.219	1.186 (0.904, 1.555)	0.219
No	0.983 (0.601, 1.607)	0.945	1.833 (1.173, 2.864)	0.008	1.031 (1.014, 1.048)	< 0.001	1.350 (1.148, 1.587)	< 0.001
Diabetes								
Yes	1.156 (0.594, 2.247)	0.670	2.118 (1.160, 3.866)	0.015	1.031 (1.008, 1.055)	0.009	1.354 (1.080, 1.699)	0.009
No	1.217 (0.717, 2.065)	0.467	2.067 (1.276, 3.347)	0.003	1.029 (1.012, 1.046)	0.001	1.325 (1.129, 1.556)	0.001

Subgroup analysis adjusted for age, ICU length of stay, cerebrovascular disease, ARDS, malignancy, PO_2_, FiO_2_, platelet count, hemoglobin, AST, ALT, GCS 24h_min_, SAPS II score, OASIS score, maximum total dose of epinephrine, and duration of epinephrine use.

BUN, blood urea nitrogen; Cr, creatinine; HR, hazard ratio; CI, confidence interval; AKI, acute kidney injury; ARDS, acute respiratory distress syndrome; PO_2_, partial pressure of oxygen; FiO_2_, fraction of inspired oxygen; AST, aspartate aminotransferase; ALT, alanine aminotransferase; GCS, Glasgow Coma Scale; SAPS II, Simplified Acute Physiology Score II; OASIS, Oxford Acute Severity of Illness Score.

For males, elevated BUN/Cr was consistently associated with higher mortality (BUN/Cr: HR = 1.024, P = 0.010; standardized BUN/Cr: HR = 1.256, P = 0.010), and T3 *vs*. T1 also reached statistical significance (HR = 1.679, P = 0.029). Among females, the associations were even stronger: BUN/Cr (HR = 1.040, P = 0.003), standardized BUN/Cr (HR = 1.357, P = 0.004), and T3 *vs*. T1 (HR = 8.091, P = 0.005) were all significant.

In AKI stage 2, BUN/Cr (HR = 1.021, P = 0.038) and standardized BUN/Cr (HR = 1.230, P = 0.038) were both associated with increased mortality, though T3 *vs*. T1 did not reach significance (P = 0.067). In stage 3, all indicators were statistically significant: T3 *vs*. T1 (HR = 2.418, P = 0.005), BUN/Cr (HR = 1.043, P < 0.001), and standardized BUN/Cr (HR = 1.508, P < 0.001).

Among patients with ARDS, higher BUN/Cr was associated with increased 1-year mortality (BUN/Cr: HR = 1.024, P = 0.003; standardized BUN/Cr: HR = 1.268, P = 0.003), and T3 group also showed significantly elevated risk (HR = 1.871, P = 0.006). These findings were similar in the non-ARDS subgroup, where continuous and standardized BUN/Cr also remained significant (HR = 1.030 and 1.333 respectively, both P = 0.015), though T3 *vs*. T1 narrowly missed significance (HR = 2.034, P = 0.064).

In patients with heart failure, neither continuous nor standardized BUN/Cr reached statistical significance (BUN/Cr: HR = 1.017, P = 0.219; standardized: HR = 1.186, P = 0.219), although T3 showed a borderline trend (HR = 2.135, P = 0.053). In contrast, among patients without heart failure, all associations were significant: T3 *vs*. T1 (HR = 1.833, P = 0.008), BUN/Cr (HR = 1.031, P < 0.001), and standardized BUN/Cr (HR = 1.350, P < 0.001).

For individuals with diabetes, higher BUN/Cr was significantly associated with increased mortality across all measures: T3 *vs*. T1 (HR = 2.118, P = 0.015), BUN/Cr (HR = 1.031, P = 0.009), and standardized BUN/Cr (HR = 1.354, P = 0.009). Similar patterns were observed in non-diabetic patients: T3 *vs*. T1 (HR = 2.067, P = 0.003), BUN/Cr (HR = 1.029, P = 0.001), and standardized BUN/Cr (HR = 1.325, P = 0.001).

### 3.5 Predictive value and dose-response relationship between BUN/Cr and mortality

**Figure 1:**
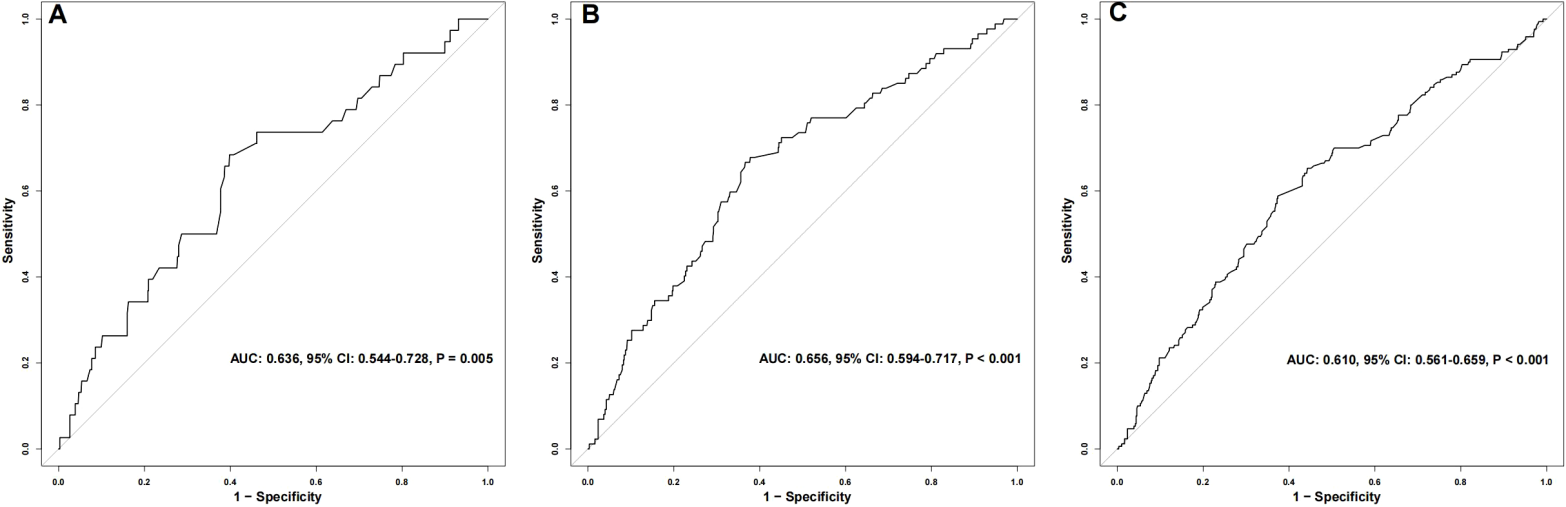
ROC curves for BUN/Cr ratio predicting 28-day **(A)**, 90-day **(B)**, and 1-year **(C)** mortality in sepsis-associated AKI patients.

**Figure 2:**
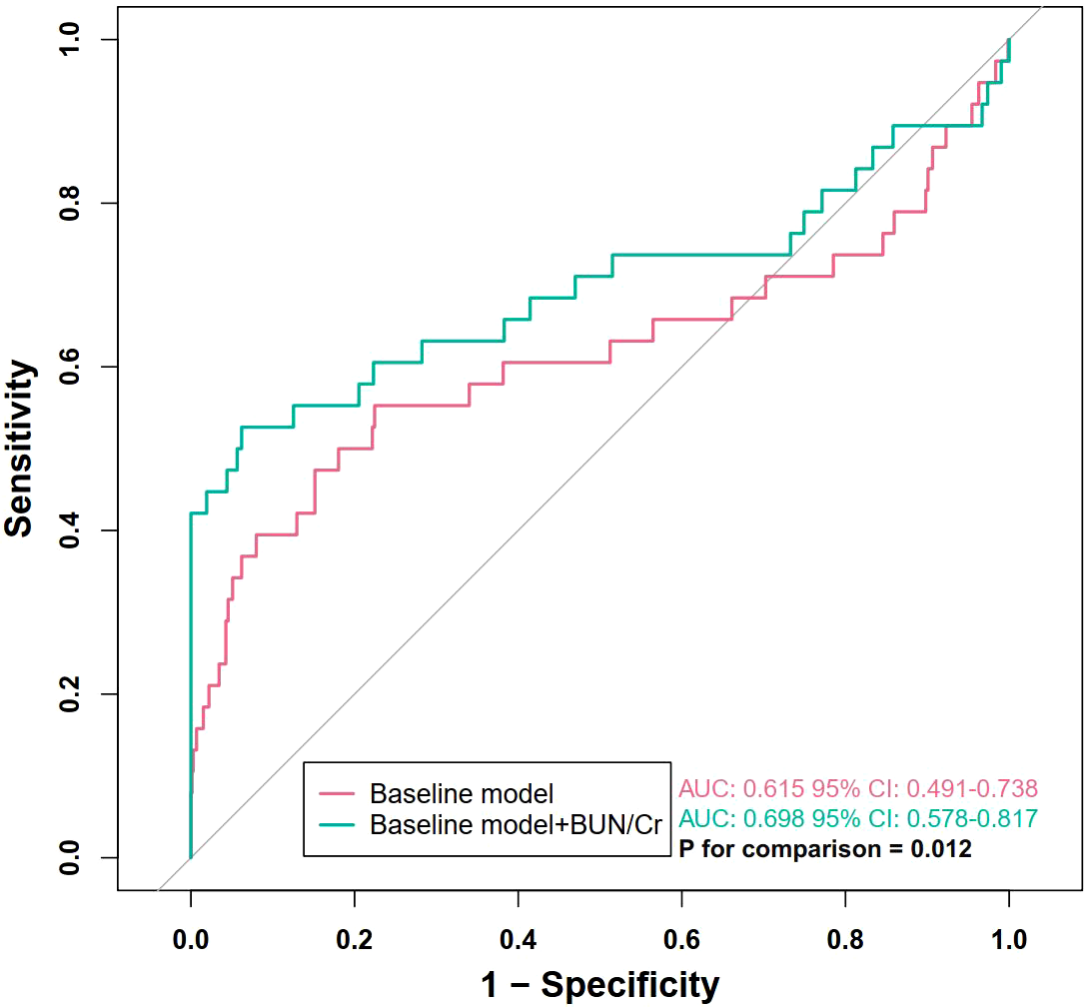
Incremental prognostic value of BUN/Cr for 28-day mortality. The baseline model included age, cerebrovascular disease, acute respiratory distress syndrome, and partial pressure of oxygen. BUN, blood urea nitrogen; Cr, creatinine. ROC, receiver operating characteristic; BUN, blood urea nitrogen; Cr, creatinine; AKI, acute kidney injury.

**Figure 3:**
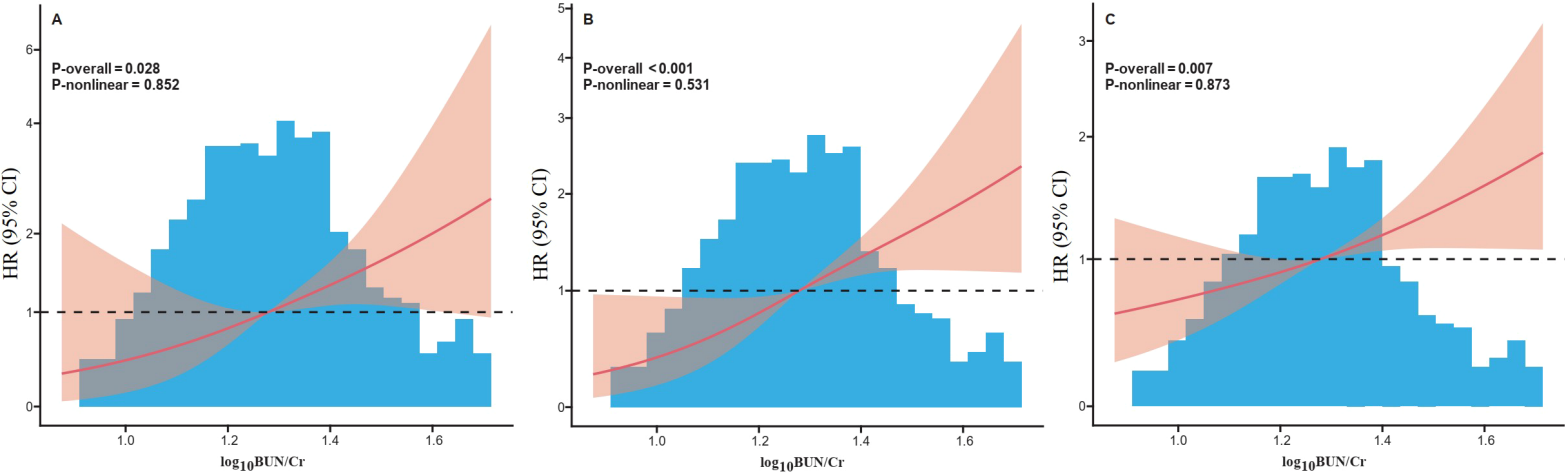
Restricted cubic spline plots showing the linear association between BUN/Cr ratio and mortality at 28 days **(A)**, 90 days **(B)**, and 1 year **(C)**. BUN, blood urea nitrogen; Cr, creatinine.

As shown in **Figure 1**, the BUN/Cr ratio demonstrated a moderate ability to predict all-cause mortality among patients with SA-AKI at 28 days, 90 days, and 1 year following discharge. The AUC was 0.636 (95% CI: 0.544–0.728, P = 0.005) for 28-day mortality, 0.656 (95% CI: 0.594–0.717, P < 0.001) for 90-day mortality, and 0.610 (95% CI: 0.561–0.659, P < 0.001) for 1-year mortality. To further evaluate whether BUN/Cr provides incremental prognostic value beyond conventional clinical predictors, a baseline model incorporating all covariates included in Model 3 was constructed for 28-day mortality. The baseline model yielded an AUC of 0.615 (95% CI: 0.491–0.738). After adding BUN/Cr to the baseline model, the AUC increased to 0.698 (95% CI: 0.578–0.817), and the difference between the two models was statistically significant (P for comparison = 0.012), indicating that BUN/Cr significantly improved the discriminatory performance of the baseline model (**Figure 2**). In **Figure 3**, RCS analyses were conducted to examine the dose-response association between the BUN/Cr ratio and mortality across the three time points. The spline curves revealed a linear relationship without significant non-linearity at 28 days (P-nonlinear = 0.852), 90 days (P-nonlinear = 0.531), or 1 year (P-nonlinear = 0.873), indicating that increases in BUN/Cr ratio were linearly associated with increased mortality risk.

## 4 Discussion

This study comprehensively evaluated the relationship between BUN/Cr and both short- and long-term all-cause mortality in patients aged 50 years and older with SA-AKI. The findings demonstrated that higher BUN/Cr levels were significantly associated with increased risks of mortality at 28 days, 90 days, and 1 year, with consistent results observed across various clinical subgroups. Additionally, ROC curve analysis showed a moderate predictive ability of BUN/Cr for mortality at each time point, while RCS analysis indicated a linear and positive association between BUN/Cr and death risk. Importantly, although the discriminative ability of BUN/Cr alone was modest, our additional analysis demonstrated that incorporation of BUN/Cr into a baseline multivariable model—including established clinical predictors adjusted in Model 3—significantly improved the model’s performance for predicting 28-day mortality. The addition of BUN/Cr increased the AUC from 0.615 to 0.698, with a statistically significant difference between models. This finding indicates that BUN/Cr provides meaningful incremental prognostic value beyond conventional clinical variables, supporting its role not merely as an isolated biomarker but as a complementary component within a comprehensive risk assessment framework. These results suggest that BUN/Cr, as a readily accessible and inexpensive biochemical marker, may enhance existing risk stratification models when integrated with established clinical parameters, particularly in resource-limited environments or where advanced risk stratification systems are not available. Its use could support early identification of high-risk individuals and facilitate timely clinical decision-making for elderly patients with SA-AKI.

Previous studies have explored the prognostic value of the BUN/Cr in various clinical settings, particularly among patients with AKI and sepsis. For example, in a retrospective study including 2,484 adult patients with septic shock, Han et al. found that the BUN/Cr ratio exhibited an “M-shaped” relationship with all-cause mortality, with patients having a BUN/Cr ≥ 27.3 showing the highest risk of death (HR = 1.596) (15). This finding suggests that BUN/Cr may serve as an independent and easily obtainable prognostic biomarker for assessing mortality risk in patients with septic shock. In another retrospective cohort study based on a nationwide population database in Taiwan including 9,717 survivors of acute kidney disease (AKD), Pan et al. used a group-based trajectory model to analyze dynamic changes in the BUN/Cr and assess its associations with major adverse kidney events (MAKEs), all-cause mortality, and major adverse cardiovascular events (MACEs) (16). The results showed that, compared with the middle-trajectory group, the high-BUN/Cr trajectory group had the greatest risks of MAKEs (HR = 1.54), mortality (HR = 1.59), and MACEs (HR = 1.57), while the low-BUN/Cr trajectory group had an increased risk of MAKEs (HR = 1.30) but a lower risk of mortality (HR = 0.84), suggesting that long-term monitoring of BUN/Cr trajectories may aid in risk stratification and prognostic assessment in patients with AKD. In addition, in a retrospective study of 665 patients hospitalized with an infection and with known pre-existing renal function, van der Slikke et al. examined the relationships among the BUN/Cr, AKI, and long-term mortality (17). The results showed that persistent AKI was closely associated with reduced long-term survival, whereas BUN/Cr was not related to transient or non-recovered AKI; however, a higher BUN/Cr identified a subgroup of patients whose long-term mortality risk was comparable to that of those with persistent AKI, suggesting that BUN/Cr may serve as an independent indicator of long-term mortality in hospitalized patients with infections rather than merely a marker of prerenal azotemia. However, in another retrospective study of 1,137 critically ill patients with cardiogenic shock (CS), Sun et al. showed that, compared with patients with a BUN/Cr < 20, those with BUN/Cr ≥ 20 had significantly better in-hospital survival (HR = 0.66), and this association remained consistent in subgroups with or without AKI, suggesting that a higher BUN/Cr may be linked to improved in-hospital prognosis in CS patients, although prospective studies are needed for confirmation (18). Additionally, in another scoping review and meta-analysis including 47 studies, Paulus et al. evaluated the value of the BUN/Cr in critical care and found that BUN/Cr was closely related to protein catabolism and persistent critical illness; moreover, a BUN/Cr ≥ 20 at ICU admission was associated with an increased risk of in-hospital mortality (relative risk = 1.60), suggesting that BUN/Cr may serve as a potential biomarker for assessing protein catabolism and prognosis in critically ill patients (19), although its use should account for confounders such as dehydration, gastrointestinal bleeding, kidney and liver dysfunction, and appropriate cut-off values should be established for different patient groups. However, although these studies have evaluated the prognostic value of BUN/Cr in patients with sepsis or AKI, its role in predicting short- and long-term mortality in patients with SA-AKI remains unclear. Unlike the above studies, the present research addresses these gaps and provides several noteworthy contributions. First, it evaluates a large cohort of 764 older adults diagnosed with SA-AKI, and assesses mortality across three distinct time points—28 days, 90 days, and 1 year—providing a more comprehensive risk profile. Second, the application of multivariable Cox proportional hazards models and RCS analysis allowed for an in-depth exploration of the linear dose-response relationship between BUN/Cr and mortality. Third, stratified analyses across key clinical subgroups—including age, sex, AKI stage, presence of ARDS, HF, and diabetes—confirmed the consistency and robustness of the findings. However, it is important to acknowledge that the predictive effect of BUN/Cr was not uniform across all subgroups. In the categorical analyses, the intermediate tertile (T2 *vs*. T1) was frequently not statistically significant, whereas the highest tertile (T3 *vs*. T1) showed a more consistent and significant increase in mortality risk. This pattern suggests a potential dose–response or threshold effect, whereby only markedly elevated BUN/Cr levels confer substantial excess risk. Moreover, in certain subgroups—such as younger patients (≤ 65 years) in the 1-year mortality analysis or patients with heart failure—the association was attenuated or not statistically significant when analyzed categorically. In contrast, when BUN/Cr was modeled as a continuous or standardized variable, the association with mortality remained significant in most subgroups, particularly for 90-day mortality. This discrepancy likely reflects differences in statistical power and the loss of information inherent to categorical stratification, rather than the absence of a biological relationship. These findings indicate that BUN/Cr should not be considered a universally strong predictor in every clinical context, but rather a marker whose prognostic strength may vary according to age, comorbidity burden, and dominant pathophysiological phenotype. In older patients and those with more severe AKI or ARDS, where systemic hypoperfusion and catabolic stress are more pronounced, the association appears more stable. Therefore, the interpretation of BUN/Cr should be individualized and integrated with the broader clinical profile rather than applied uniformly across all subgroups. Fourth, beyond evaluating BUN/Cr as an isolated predictor, we further examined its incremental prognostic value by integrating it into a multivariable baseline model composed of established clinical risk factors. The significant improvement in model discrimination after incorporating BUN/Cr highlights its additive value within a comprehensive risk assessment framework, thereby strengthening its clinical applicability. Finally, the standardization of BUN/Cr facilitated quantitative interpretation and improved the clinical comparability of results. Together, these strengths position this study ahead of many existing reports and lay a solid foundation for future clinical research and practice.

In older (≥ 50 years) patients with SA-AKI, an elevated BUN/Cr ratio reflects multiple converging mechanisms that portend worse short- and long-term outcomes. Importantly, BUN/Cr should not be interpreted as a purely renal marker in the context of sepsis. Rather, it represents a composite indicator influenced by systemic hemodynamic, hormonal, inflammatory, and metabolic alterations. Severe systemic inflammation and stress in sepsis provoke a hypercatabolic state, accelerating proteolysis and muscle breakdown; consequently, urea production surges while muscle mass and creatinine generation decline (20). This combination raises the BUN/Cr ratio and reflects significant muscle wasting (sarcopenia), a marker of frailty in older patients (21). Impaired renal perfusion from septic shock further elevates the ratio: hypovolemia activates the renin–angiotensin system, enhancing urea reabsorption and driving prerenal azotemia (2, 22, 23). In addition, increased secretion of antidiuretic hormone (ADH) during sepsis promotes enhanced tubular urea reabsorption in the collecting ducts, further elevating BUN levels independently of glomerular filtration changes. Alterations in renal blood flow distribution and tubular transport function in sepsis may also contribute to disproportionate changes in urea and creatinine handling. Either extreme of fluid imbalance can compound this risk. Inadequate resuscitation causes dehydration and prerenal azotemia, whereas fluid overload in sepsis fosters edema and can mask rising creatinine, reflecting deranged perfusion – both scenarios are linked to higher mortality (24). In addition, several clinical factors unrelated to intrinsic renal injury may substantially influence the BUN/Cr ratio. Fluid balance status, including both dehydration and fluid overload, may alter serum urea concentration independently of glomerular filtration. Nutritional status and dietary protein intake directly affect urea production, while gastrointestinal bleeding increases protein absorption from digested blood, thereby elevating BUN levels. Furthermore, medications such as corticosteroids may enhance protein catabolism, and diuretics may modify volume status and renal hemodynamics, indirectly affecting the ratio. These factors highlight that BUN/Cr is a multifactorial and nonspecific marker that should be interpreted within the broader systemic context of critical illness. Furthermore, SA-AKI often represents a spectrum of injury that includes pre-renal hypoperfusion, intrinsic tubular damage, and mixed forms of injury. These different pathophysiological phenotypes may exert distinct effects on BUN and creatinine kinetics, leading to variability in the BUN/Cr ratio. Therefore, an elevated BUN/Cr in sepsis may reflect not only renal dysfunction per se, but also systemic hypoperfusion, neurohormonal activation, inflammatory burden, and enhanced protein catabolism. These pathophysiologic pathways help explain why a high BUN/Cr ratio is a strong predictor of mortality in sepsis.

Interestingly, our baseline data (**Table 1**) revealed that the T1 group (lower BUN/Cr) exhibited higher SOFA scores, a greater proportion of AKI stage III, and more frequent use of renal replacement therapy, whereas the T3 group showed characteristics more suggestive of hypoperfusion and hypercatabolic states. At first glance, this apparent discrepancy may seem to complicate the interpretation of the association between BUN/Cr and mortality. However, this pattern further supports the concept that BUN/Cr reflects distinct pathophysiological phenotypes rather than merely the overall severity of organ failure. Specifically, a lower BUN/Cr in the T1 group may largely reflect markedly elevated creatinine due to severe reductions in glomerular filtration rate, while a higher BUN/Cr in the T3 group may indicate relatively preserved creatinine levels in the setting of systemic hypoperfusion, neurohormonal activation, ADH-mediated tubular urea reabsorption, and enhanced protein catabolism. Therefore, BUN/Cr may capture risk pathways driven by circulatory dysfunction and metabolic stress that are not fully represented by conventional severity scores such as SOFA or AKI stage. This heterogeneity underscores the importance of interpreting BUN/Cr within the broader clinical and pathophysiological context rather than labeling specific tertiles as simply “low-risk” or “high-risk”.

Although this study was carefully designed and employed robust statistical methods, several limitations should be acknowledged when interpreting the findings. First, this was a single-center, retrospective cohort study. Despite a relatively adequate sample size and extended observation period, center-specific factors—such as institutional protocols, critical care management strategies, and patient demographics—may limit the generalizability of the results. Multicenter, prospective studies with larger populations are needed to validate these findings. Second, BUN and serum Cr are common but non-specific clinical markers that can be influenced by various non-pathological factors, including dietary protein intake, hydration status, and muscle mass. This is particularly relevant in older adults, where Cr levels may underestimate renal dysfunction, potentially impacting the accuracy of the BUN/Cr ratio and its prognostic interpretation. Moreover, detailed data regarding cumulative fluid balance, precise nutritional intake, gastrointestinal bleeding burden, and comprehensive medication exposure (e.g., corticosteroids or diuretics) were not consistently available in this retrospective dataset. As a result, these potentially influential variables could not be fully incorporated into the multivariable models, and residual confounding related to these factors cannot be excluded. Third, although multivariable Cox proportional hazards regression was used to adjust for a wide range of potential confounders and subgroup analyses confirmed the stability of associations, residual or unmeasured confounding remains possible. Specifically, key variables reflecting systemic inflammation and nutritional status were not consistently available in this retrospective dataset and therefore could not be incorporated into the models, despite their potential relevance to both BUN/Cr levels and clinical outcomes. Similarly, in-hospital therapeutic strategies (e.g., antimicrobial escalation, fluid resuscitation decisions, or dialysis initiation timing) were often recorded as unstructured text and could not be standardized for analysis. We recognize these factors may influence patient outcomes and plan to address them in future studies using structured data extraction or natural language processing techniques. Fourth, BUN/Cr was assessed only once within the first 24 hours after sepsis diagnosis. Dynamic changes in this ratio over time, which may better reflect disease progression or recovery, were not captured. Consequently, the prognostic implications of longitudinal BUN/Cr trajectories could not be evaluated in the present study. Future studies should consider serial measurements and time-series modeling to enhance predictive accuracy. Fifth, although differentiating between pre-renal and intrinsic renal mechanisms of SA-AKI could offer important pathophysiological insights, neutrophil gelatinase-associated lipocalin (NGAL), a sensitive biomarker of tubular injury, was not routinely measured in our institution during the study period and was therefore absent from existing inpatient records. In addition, most patients did not undergo systematic 24-hour urine collection during the early phase of sepsis (within 24 hours of diagnosis), and the available urine sodium measurements were largely sporadic spot samples with inconsistent timing and documentation. As a result, we were unable to evaluate tubular injury–specific biomarkers or to assess dynamic urinary sodium excretion, limiting our ability to distinguish AKI subtypes or to explore mechanistic pathways underlying the observed association between BUN/Cr and mortality. Sixth, this study used all-cause mortality as the primary endpoint without further classification of causes of death. Therefore, it remains unclear whether the BUN/Cr ratio is more strongly associated with specific outcomes such as multiorgan failure, infection-related death, or cardiovascular events. Additionally, follow-up data were obtained from electronic medical records and telephone interviews, which may be subject to missing information or misclassification. Seventh, although RCS analysis in the overall cohort supported a linear association between BUN/Cr and mortality, subgroup analyses demonstrated heterogeneity in effect size, with the association attenuated or not statistically significant in certain subgroups (e.g., younger patients or those with heart failure). This suggests that the linear trend observed at the population level may not uniformly apply across all clinical phenotypes. The predictive strength of BUN/Cr may therefore depend on underlying patient characteristics and dominant pathophysiological mechanisms. In addition, subgroup analyses are inherently subject to reduced statistical power and wider confidence intervals, which may partly explain the lack of significance in some strata. Consequently, the assumption of linearity should be interpreted primarily at the overall cohort level rather than as universally applicable to every subgroup. Despite these limitations, we attempted to minimize bias by including multiple validated illness severity scores (e.g., SOFA, SAPS II, OASIS), oxygenation indices, hemoglobin, and hepatic/renal function markers in the multivariable model. Furthermore, BUN/Cr was modeled both as a continuous and standardized variable, as well as across tertiles, to ensure consistency and reliability of interpretation. In conclusion, while this study provides important prognostic insights into the utility of the BUN/Cr ratio in older patients with SA-AKI, the results should be interpreted with caution. Future research should adopt multicenter, prospective designs and explore integration with dynamic monitoring, additional biomarkers, and multimodal machine learning approaches to enhance clinical applicability and explanatory value.

## 5 Conclusion

In summary, this study is the first to comprehensively examine the association between BUN/Cr and both short- and long-term all-cause mortality in a large cohort of older adults with SA-AKI. The findings demonstrate that BUN/Cr is an independent predictor of mortality, with a measurable predictive value and a clear linear dose–response relationship. Importantly, BUN/Cr should not be interpreted as a direct surrogate of renal injury alone; rather, it likely represents a composite marker reflecting multiple pathophysiological processes associated with sepsis severity, including alterations in perfusion status, neurohormonal activation, protein catabolism, and tubular function.

Given its availability and simplicity as part of routine laboratory testing, BUN/Cr may serve as a practical tool for early risk stratification in clinical settings. It can aid clinicians in identifying high-risk patients promptly, thereby supporting more timely and targeted management strategies. Future research should further investigate the respective contributions of systemic perfusion, metabolic catabolism, and tubular transport dysfunction to variations in BUN/Cr, in order to better elucidate its mechanistic basis and refine its prognostic application. Moreover, future research should explore how BUN/Cr can be integrated with existing risk scores or novel biomarkers to develop multidimensional prognostic models, with the aim of enhancing personalized treatment approaches in this vulnerable population.

## Data Availability

The raw data supporting the conclusions of this article will be made available by the authors, without undue reservation.
